# Anatomical and Functional Characterization of Central Amygdala Glucagon-Like Peptide 1 Receptor Expressing Neurons

**DOI:** 10.3389/fnbeh.2021.724030

**Published:** 2021-12-24

**Authors:** Ningxiang Zeng, Elam J. Cutts, Christian B. Lopez, Simran Kaur, Miguel Duran, Sonja A. Virkus, J. Andrew Hardaway

**Affiliations:** Department of Psychiatry and Behavioral Neurobiology, University of Alabama at Birmingham, Birmingham, AL, United States

**Keywords:** central amygdala (CeA), anatomy, neural circuit, electrophysiology, glucagon-like peptide 1 receptor (GLP-1R), glucagon-like peptide 1 (GLP-1)

## Abstract

Glucagon-like peptide 1 receptors (GLP-1Rs) are highly expressed in the brain and are responsible for mediating the acute anorexigenic actions of widely prescribed GLP-1R agonists. Neurobiological efforts to localize the hypophagic effects of GLP-1R agonists in the brain have mainly focused on the hypothalamus and hindbrain. In this study, we performed a deep anatomical and neurophysiological characterization of GLP-1Rs in the central nucleus of the amygdala (CeA). At an mRNA level, we found that *Glp1r* is diffusely coexpressed in known CeA subpopulations like protein kinase c δ (*Prkcd*), somatostatin (*Sst*), or tachykinin2 (*Tac2*). At a cellular level, we used *Glp1r-*Cre mice and viral Cre-dependent tracing to map the anatomical positions of GLP-1R cells across the rostral-caudal axis of the CeA and in CeA subdivisions. We found that *Glp*1*r*^CeA^ cells are highly enriched in the medial subdivision of the CeA (CeM). Using whole cell patch clamp electrophysiology, we found that *Glp*1*r*^CeA^ neurons are characterized by the presence of I_h_-like currents and resemble a low threshold bursting neuronal subtype in response to hyperpolarizing and depolarizing current injections. We observed sex differences in the magnitude of I_h_-like currents and membrane capacitance. At rest, we observed that nearly half of *Glp*1*r*^CeA^ neurons are spontaneously active. We observed that active and inactive neurons display significant differences in excitability even when normalized to an identical holding potential. Our data are the first to deeply characterize the pattern of *Glp1r* in the CeA and study the neurophysiological characteristics of CeA neurons expressing *Glp1r*. Future studies leveraging these data will be important to understanding the impact of GLP-1R agonists on feeding and motivation.

## Introduction

Obesity is a growing health problem for developed nations. In the United States alone, the CDC estimated that obesity prevalence among adults increased from 30.5 to 42.4% over the last 18 years indicating more than a third of adults in the United States are obese (BMI > 30). With obesity comes an increased risk for chronic diseases including cardiovascular disease, cancer, and type II diabetes. Many individuals with type II diabetes are prescribed drugs that target the glucagon-like peptide 1 receptor (GLP-1R) due to the clinical benefit of enhancing the release of insulin while also decreasing appetite (Müller et al., 2019). Despite their wide use, a complete understanding of the full biological and behavioral impacts of GLP-1R agonism is lacking.

When administered *via* intracerebroventricular (icv) injection, GLP-1R agonists elicit hypophagia indicating that neuronal GLP-1Rs are required for this hypophagic effect. Thus, the anorexigenic effect of GLP-1R agonists can be attributed to the activation of neuronal not peripheral GLP-1Rs (Burmeister et al., 2013, 2017; Secher et al., 2014; Sisley et al., 2014; Adams et al., 2018). Attempts to localize and attribute the actions of GLP-1R signaling on feeding to specific brain nuclei have focused mainly on hypothalamic and hindbrain GLP-1Rs known to control hunger states or meal termination (Hayes et al., 2009; Kanoski et al., 2011; Alhadeff et al., 2014). However, the function of GLP-1Rs in limbic sites—like the lateral septum (LS), bed nucleus of the stria terminalis (BNST), and the central amygdala (CeA)—likely plays a key motivational role in feeding. Of relevance, recent work has shown that BNST GLP-1Rs and GLP-1R-expressing cells may in part drive stress-induced hypophagia and that LS GLP-1Rs functionally regulate feeding and motivation (Terrill et al., 2016; Williams et al., 2018). In the CeA, however, very little is known about how GLP-1Rs regulate either feeding or motivation.

The CeA is a complex heterogenous structure that contains both orexigenic and anorexigenic subpopulations of neurons. These neuronal subpopulations regulate emotional and survival behaviors including responses to predators and noxious stimuli (Haubensak et al., 2010; Li et al., 2013; Janak and Tye, 2015; Douglass et al., 2017; Hardaway et al., 2019; Ip et al., 2019; Steinberg et al., 2020; Weera et al., 2021). Previous studies using mice and rats have identified the CeA as a site for GLP-1R expression; however, the pattern of GLP-1R expression within a framework of known CeA populations has been unclear. In this study, we performed a deep anatomical characterization of CeA GLP-1Rs and GLP-1R-expressing neurons and utilized slice electrophysiology to characterize the membrane and neurophysiological properties of *Glp*1*r*^CeA^ neurons.

## Materials and Methods

### Animals

We used 8–16 week old C57BL6/J (Jackson Labs, Bar Harbor, ME) or *Glp1r-*Cre (MGI ID: 5776617) male and female mice for this study (Williams et al., 2016). For all experiments, animals were group housed 3–5/cage. Mice were maintained on a standard 12:12 light cycle with lights on at 7 a.m. Unless otherwise indicated, food and water were provided *ad libitum*. All procedures were performed according to an approved animal protocol by the University of Alabama at Birmingham Institutional Animal Care and Use Committee.

### Stereotaxic Surgery

Survival surgeries were performed on 6–8 week old *Glp1r-*Cre mice. On the day of the surgery, animals received 0.1 mg/kg Buprenorphine SR (sc) and 5 mg/kg Meloxicam SR (sc). Under isoflurane inhalation (0.5–5%), mice were placed in a stereotaxic frame. The scalp was depilated and sterilized using rotating 70% EtOH and betadine application. Topical lidocaine (4%) and triple antibiotic were then applied to the scalp in preparation for incision. After ensuring a deep plane of anesthesia *via* loss of toe pinch reflex, a midline incision was made to expose the skull surface. A craniotomy was then performed at the injection site above the CeA. AAV5-hSyn-DIO-mCherry (300 nl, Addgene - lot# v63478) was injected at −1.30 mm posterior, ±2.90 mm lateral, and −4.60 mm ventral of bregma. Mice remained in the colony for 3–4 weeks following surgery for post-operative recovery and to allow accumulation of mCherry expression in the CeA prior to perfusion or electrophysiological recordings.

### Histology

*Glp1r-*Cre mice were injected with a terminal dose of tribromoethanol (250 mg/kg). After reaching a deep plane of anesthesia confirmed *via* loss of toe pinch reflex, animals were transcardially perfused with 0.01 M PBS and then 50 ml of 4% paraformaldehyde in 0.01 M PBS. Brains were extracted and post-fixed in 4% paraformaldehyde/PBS overnight then transferred to 30% sucrose for cryoprotection for 36–48 h prior to cryosectioning. Free floating sections were mounted to Superfrost plus microscope slides (Fisher Scientific; Waltham, MA) and coverslipped with mounting media containing DAPI (Vector Laboratories).

### Fluorescence *in situ* Hybridization

Fresh tissue was harvested from 8 to 16 week old C57BL6J or *Glp1r*-Cre male and female mice and flash frozen on dry ice then stored at −80°C. Brains were cryosectioned at 20 μM and directly mounted onto Superfrost Plus slides (Fisher Scientific; Waltham, MA) then stored in a sealed container at −80°C. The single molecule RNAscope fluorescent multiplex assay was performed according to the manufacturer's protocol (Advanced Cell Diagnostics; Newark, CA). The target probe for *Glp1r* was applied in tandem with the appropriate probe for *Prkcd, Sst, Tac2*, or *Cre*. For each RNAscope multiplex florescent assay, the target probe for *Glp1r* was assigned to 647 nm excitation while target probes for *Prkcd, Sst, Tac2*, and *Cre* were assigned to 550 nm excitation using the appropriate amplification buffers. Appropriate negative controls from the manufacturer were included for each assay for verification of probe specificity. The following target probes were used for this manuscript:

**Table:** 

446391-Mm-*Tac2*	Accession #: NM_009312.2
404631-Mm-*Sst*	Accession #: NM_009215.1
441791-Mm-*Prkcd*	Accession #: NM_011103.3
418851-C3-Mm-*Glp1r*	Accession #: NM_021332.2
312281-*Cre*	Accession #: KC845567.1

### Imaging and Image Analysis

Imaging was performed on a Keyence BZ-X810 under 20X magnification. *Glp1*r was imaged with 647 nM to maximize sensitivity of detection. For tissue sections obtained from C57BL6/J mice, tiled z-stacks of the CeA and surrounding region were captured using optical sectioning. For tissue sections obtained from *Glp1r*-Cre mice, tiled z-stack images of the CeA and surrounding regions were captured using widefield florescence imaging. Raw images were then stitched and a maximum intensity projection made in Keyence Analyzer. Stitched raw images were obtained for individual channels including DAPI, 647, 550, and 488 nm. Prior to creating composite images, non-specific background signal was removed through image subtraction of images obtained on 488 nm from those obtained on 647 and 550 nm. No probes were developed using 488 nm fluorophores. Composite images were then created and processed for quantitative analysis. Cell counts and area measurements were performed in ImageJ (Fiji). Final images were assembled in Adobe Illustrator 25.2.3.

For quantitative analysis of *Glp1r* expressing neurons across the rostral-caudal axis, three subsections were defined with respect to bregma coordinates as determined using The Mouse Brain in Stereotaxic Coordinates, 4th ed. (Franklin and Paxinos, 2019). The range for anterior, middle, and posterior CeA were defined as follows: the anterior CeA falls within 0.71–1.03 mm posterior of bregma, middle CeA falls within 1.03–1.43 mm posterior of bregma, and posterior CeA falls within 1.43–1.79 mm posterior of bregma. Following acquisition, images were categorized as either anterior, middle, or posterior for quantitative analysis. Classification was determined using anatomical landmarks including the overall size of the CeA, the size and width of the basolateral amygdala, the position of the intercalated cells, and the presence and shape of the stria terminalis. Count data for this analysis was obtained from 6 to 8 images per mouse (2 male and 2 female) with 3–4 images of sections ~100 μM apart with respect to bregma acquired per hemisphere per mouse.

### Patch Clamp Electrophysiology

#### Slice Preparation

For electrophysiology, animals were removed from their cage and brought to the lab for brain slice preparation. The animal rested in a quiet chamber for 30–45 min prior to slice preparation to dissipate stress associated with animal transport from the vivarium. Mice were treated with a lethal dose of tribromoethanol (250 mg/kg, i.p.), and, after a deep plane of anesthesia was reached, animals were transcardially perfused with cold, sodium free N-methyl-D-glucamine (NMDG) artificial cerebrospinal fluid (aCSF) [(in mM) 93 N-methyl-D-glucamine, 2.5 KCl, 1.2 NaH_2_PO_4_, 30 NaHCO_3_, 20 HEPES, 25 Glucose, 5 L-ascorbic acid, 2 Thiourea, 3 sodium pyruvate, 10 MgSO_4_ X 7H_2_O, 0.5 CaCl_2_ X 2H_2_O]. All solutions were saturated with 95% CO_2_ and 5% O_2_. The brain was rapidly dissected and coronal 300 μM sections prepared in ice cold, oxygenated NMDG aCSF using a Leica VT1200S at 0.07 mm/s. Slices were immediately transferred to 32°C NMDG aCSF for 10 min, and then normal 32°C aCSF [(in mM): 124 NaCl, 4.4 KCl, 2 CaCl_2_, 1.2 MgSO_4_, 1 NaH_2_PO_4_, 10.0 glucose, and 26.0 NaHCO_3_]. Slices rested in normal aCSF for at least 30 min prior to recordings.

#### Recording

Whole cell patch clamp recordings were performed in the CeA guided by DIC microscopy and mCherry fluorescence. Slices were then transferred to a recording chamber (Warner Instruments), submerged in normal, oxygenated aCSF and maintained at 32°C with a flow rate of 2 ml/min. We patched mCherry-expressing neurons in a balanced fashion in all CeA subdivisions in which they were present. For all recordings we used a potassium gluconate internal solution [(in mM): 135 C_6_H_11_KO_7_, 5 NaCl, 2 MgCl_2_, 20 HEPES, 0.6 EGTA, 4 Na_2_ATP, 0.4 Na_2_GTP at a final osmolarity of 290 mOsm at a pH of 7.3]. For voltage clamp recordings, neurons were voltage clamped at −70 mV using a Multiclamp 700B and currents were digitized with an Axon 1550B digitizer (Molecular Devices, Fremont, CA).

#### Data Analysis

Data were analyzed in Clampfit 11.1 (Molecular Devices, San Jose, CA). Membrane capacitance and resistance were determined online using a −10 mV square pulse. We did not correct for liquid junction potential. Resting membrane potential was determined using a 2' gap free current clamp recording where we took the average following a stabilizing period (usually 30”). For active neurons, we counted the number of action potentials during this short recording to determine the average spiking frequency. For rheobase, we identified the injected current at the peak of the first action potential. For recordings at −70 mV, we injected a steady variable amount of negative current using the amplifier while recording gap-free in current clamp until the cell reached −70 mV. For determination of spiking subtype, we identified whether the cells had rebound spikes following a negative current injection, the latency to first spike, and if a cell had spike frequency adaptation. Late spiking neurons had a latency to first spike of >100 ms. Regular spiking neurons had a latency < 100 ms and minimal spike frequency adaptation. Low threshold bursting neurons had a short latency to the first spike, reduced spike frequency following the first 1–3 action potentials, and had a characteristic rebound spike. For detection of I_h_-like currents, we measured the difference in cell current immediately following a negative voltage step to the point of stable current (at least 250 ms into a 500 ms voltage step). We measured the difference at each voltage step. To determine the proportion of neurons that demonstrated I_h_-like currents we used a threshold of −20 pA at the most hyperpolarized voltage step (−120 mV). Any cell with < −20 pA at −120 mV was empirically categorized as lacking I_h_-like current.

### Statistics

All statistical analyses were performed using Graphpad Prism version 9.0.0. For electrophysiology data, original data were analyzed in Clampfit 11.1. Figures were assembled in Adobe Illustrator.

## Results

**Figure 1:**
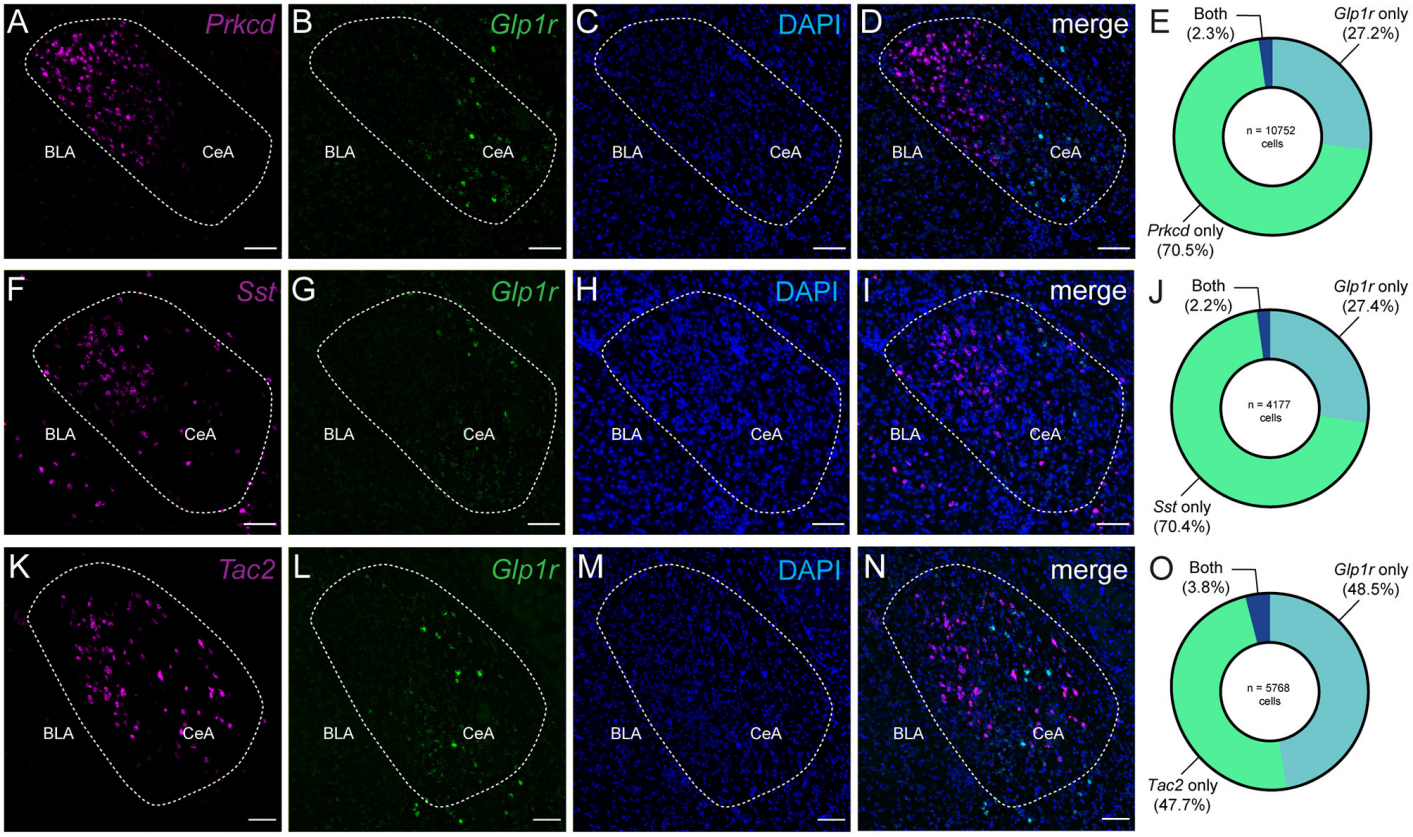
The Glucagon-like peptide 1 receptor is expressed diffusely amongst known genetic markers of the central amygdala. **(A–D)** Example single channel and merged images of *Prkcd, Glp1r*, and DAPI mRNA in the CeA of wild type mice. **(E)** Donut plot depicting percentages of *Prkcd, Glp1r*, and double-labeled cells. **(F–I)** Example single channel and merged images of *Sst, Glp1r*, and DAPI mRNA in the CeA of wild type mice. **(J)** Donut plot depicting percentages of *Sst, Glp1r*, and double-labeled cells. **(K–N)** Example single channel and merged images of *Tac2* and *Glp1r* mRNA, and DAPI in the CeA of wild type mice. **(O)** Donut plot depicting percentages of *Tac2, Glp1r*, and double-labeled cells. For **(E,J,O)**, count data were obtained from 6 to 8 images acquired from 4 wildtype C57BL6/J mice (2 male and 2 female) with 3–4 images per hemisphere per mouse. **(E)** A total of 10,752 cells were counted with 2,688 ± 558 (s.e.) cells counted per animal. **(J)** A total of 4,177 cells were counted with 1,044 ± 82.2 (s.e.) cells counted per animal. **(O)** A total of 5,788 cells were counted with 1,442 ± 229 (s.e.) cells counted per animal. Scale bar = 100 μm for all images.

Previous murine studies have demonstrated the presence of GLP-1Rs in the CeA (Cork et al., 2015; Graham et al., 2020); however, we sought to characterize the pattern of GLP-1R expression within a framework of known genetic markers of the CeA. To that end, we used fluorescence *in situ* hybridization in the CeA to characterize *Glp1r* mRNA. In our first experiment, we found that *Glp1r-*containing cell bodies did not coexpress *Prkcd*, a member of the protein kinase c family that is highly enriched in the lateral and central subdivisions of the CeA (Figures 1A–E) (Haubensak et al., 2010). Of the total neurons counted (*n* = 10,752 cells, 2,688 ± 558 cells per mouse, 4 mice), only 2.3% expressed both *Prkcd* and *Glp1r*. Notably, the CeL also has an abundant cell group that express the neuropeptide somatostatin (*Sst*) (Li et al., 2013), and these neurons do not coexpress *Prkcd*. Together, *Sst* and *Prkcd* make up the dominant, nearly exclusive cell types in the CeL (McCullough et al., 2018b). As with *Prkcd*, we observed only a small population of *Glp1r*+ neurons coexpress *Sst* (2.2%) with respect to the total number of cells counted (*n* = 4,177 cells, 1,044 ± 82.2 cells per mouse, 4 mice) (Figures 1F–J). In both experiments, we observed that *Glp1r* transcripts were enriched in cell bodies of the medial subnucleus of the CeA (CeM); therefore, we selected *Tac2*, a neuropeptide whose expression is more enriched in the CeM. Interestingly, *Glp1r* was not robustly coexpressed with cells that express *Tac2* with only 3.8% of total neurons counted (*n* = 5,768 cells, 1,442 ± 229 cells per mouse, 4 mice) showing coexpression (*Glp1r*+*/Tac2*+) (Figures 1K–O) (Andero et al., 2014). These data are consistent with a diffuse coexpression pattern of *Glp1r* within known genetically-defined populations of the CeA.

**Figure 2:**
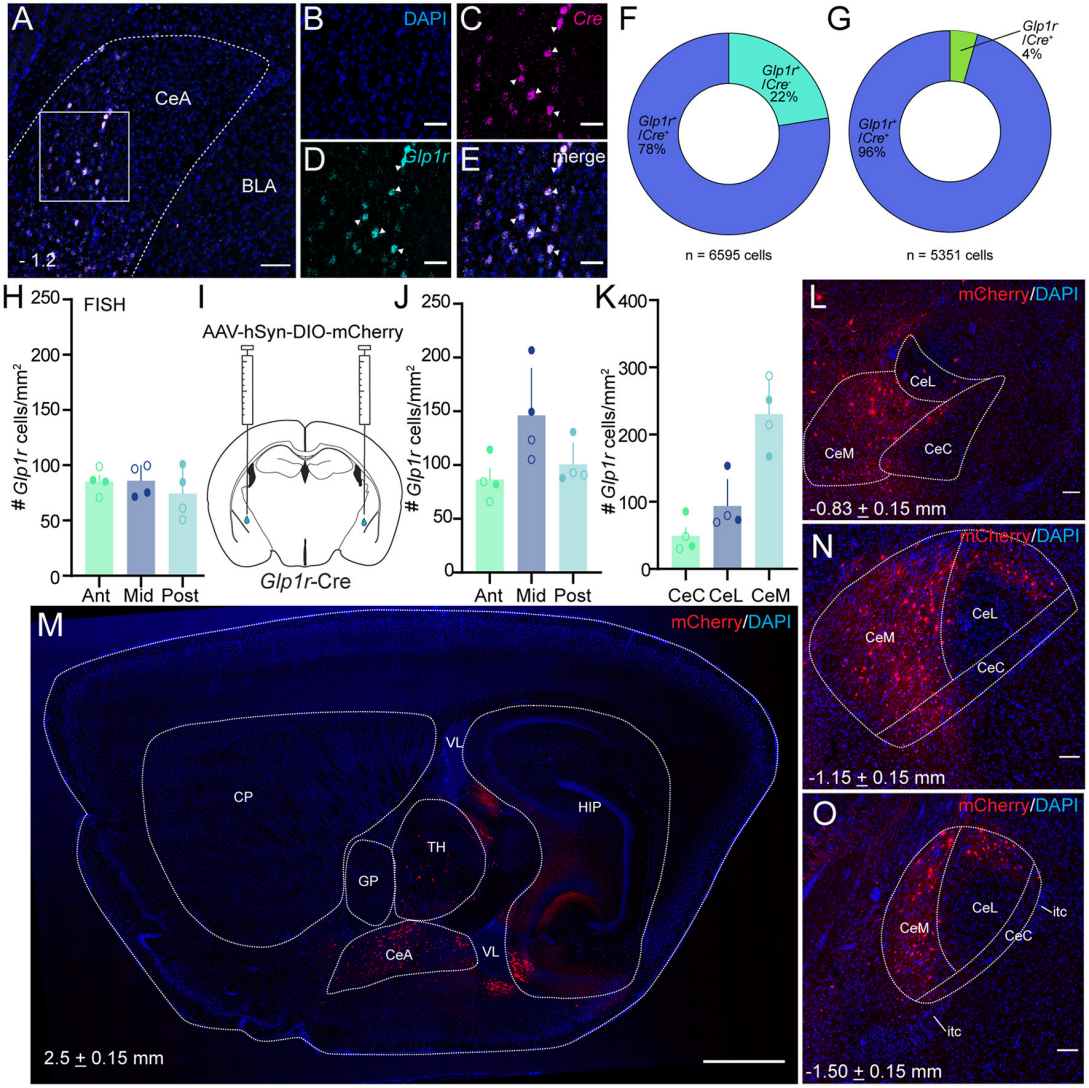
*Glp*1*r*^CeA^ neurons are distributed across the rostral-caudal axis and enriched in the CeM. (**A–G**) *Glp1r*-Cre line validation using dual florescence *in situ* hybridization. **(A)** Wide field merged view of CeA with *Glp1r* in cyan and *Cre* in magenta. Square represents enlarged, single channel view shown in **(B–D)** and enlarged merged view in **(E)**. **(F)** Quantification of *Cre* mRNA expression within *Glp1r*+ neurons (*n* = 6,595 cells, 824.4 ± 125.6 s.e. cells per mouse). **(G)** Quantification of *Glp1r* mRNA expression within *Cre*+ neurons (*n* = 5,351 cells, 668.9 ± 101.3 s.e. cells per mouse). Data shown in **(F,G)** was obtained from 8 images taken from 4 mice. **(H)** Schematic of CeA injection of AAV-hSyn-DIO-mCherry in *Glp1r*-Cre mice. **(I)** Quantification of FISH labeled *Glp*1*r*^CeA^ neurons across the rostral-caudal axis (counts from 3 to 4 sections per subdivisions per mouse). **(J)** Quantification of virally tagged *Glp1r*^*CeA*^ neurons across the rostral-caudal axis [*n* = 4, including two male and two female wild type mice; Ordinary one-way ANOVA with Tukey's multiple comparisons test; *f* = 4.190, *p* = 0.0520 (Ant vs. Mid), 0.7997 (Ant vs. Post) and 0.1402 (Mid vs. Post)]. **(K)** Quantification of *Glp1r*^*CeA*^ neurons at different subdivisions of CeA [*n* = 4, including two male and two female wild type mice; Ordinary one-way ANOVA with Tukey's multiple comparisons test; *f* = 21.89, *p* = 0.3125 (CeC vs. CeL), 0.0004 (CeC vs. CeM) and 0.0025 (CeL vs. CeM)]. For **(H–K)**, open circles represent data obtained from female mice and closed circles represent data obtained from male mice. **(L,N,O)** Example coronal images of *Glp*1*r*^CeA^ distribution in anterior **(L)**, middle **(N)** and posterior **(O)** segments of the CeA. **(M)** Example sagittal image of *Glp*1*r*^CeA^ distribution. Note some viral tagging of putative *Glp1r* neurons outside the CeA. Scale bars represent 100 μm **(A,M)** or 50 μm **(B–E)**. For all experiments, *p < 0.05, **p < 0.01, and ***p < 0.001. CP, caudoputamen; GP, globus pallidus; TH, thalamus; VL, lateral ventricle; HIP, hippocampal region.

While amplification-based *in situ* hybridization is an effective strategy for mRNA detection, we used an alternative cell-labeling approach to quantify the pattern of *Glp1r* cell distribution in the CeA. In order to validate the *Glp1r*-Cre mouse line, we employed florescence *in situ* hybridization techniques to assess the penetrance and fidelity of *Cre* expression through colocalization analysis of *Glp1r* and *Cre* mRNA (Figures 2A–G). Of the *Glp1r*+ cells counted (*n* = 6,595 cells, 824.4 ± 125.6 s.e. cells per mouse), 77.6% expressed *Cre* demonstrating high penetrance of this transgenic mouse strain. Additionally, 95.6% of total *Cre*+ cells counted (*n* = 5,351 cells, 668.9 ± 101.3 s.e. cells per mouse) coexpressed *Glp1r* confirming high fidelity. Together, these data verify the validity of the *Glp1r*-Cre mouse. Using *Glp1r*-Cre mice, we stereotaxically injected AAV-DIO-mCherry into the CeA (Figure 2I) which produced an expression pattern of mCherry that resembled native *Glp1r* mRNA. Using these mice, we serially mounted and counted *Glp*1*r*^CeA^ cells across the rostral-caudal axis and observed *Glp*1*r*^CeA^ cells throughout the entire range of the CeA and outside, but proximal to the CeA (Figure 2M). In response to this finding, we performed careful cell counts across three equal A-P segments and observed a higher density of *Glp*1*r*^CeA^ cells in the middle third of the CeA; however, this did not reach statistical significance (Figure 2J). Similarly, quantification of *Glp1r*-expressing cells at the mRNA level across the three segments of the CeA revealed no difference in the density of *Glp1r*-expressing cells (Figure 2H). We then used anatomical landmarks and atlases to count *Glp1r*+ cells in the lateral, capsular, and medial subdivisions of the CeA and identified a significant enrichment in the density of *Glp*1*r*^CeA^ cells in the CeM (Figures 2K,L,N,O). Thus, *Glp*1*r*^CeA^ cells are expressed throughout the rostral-caudal axis of the CeA and enriched in the output subnucleus of the CeA - the CeM.

**Figure 3:**
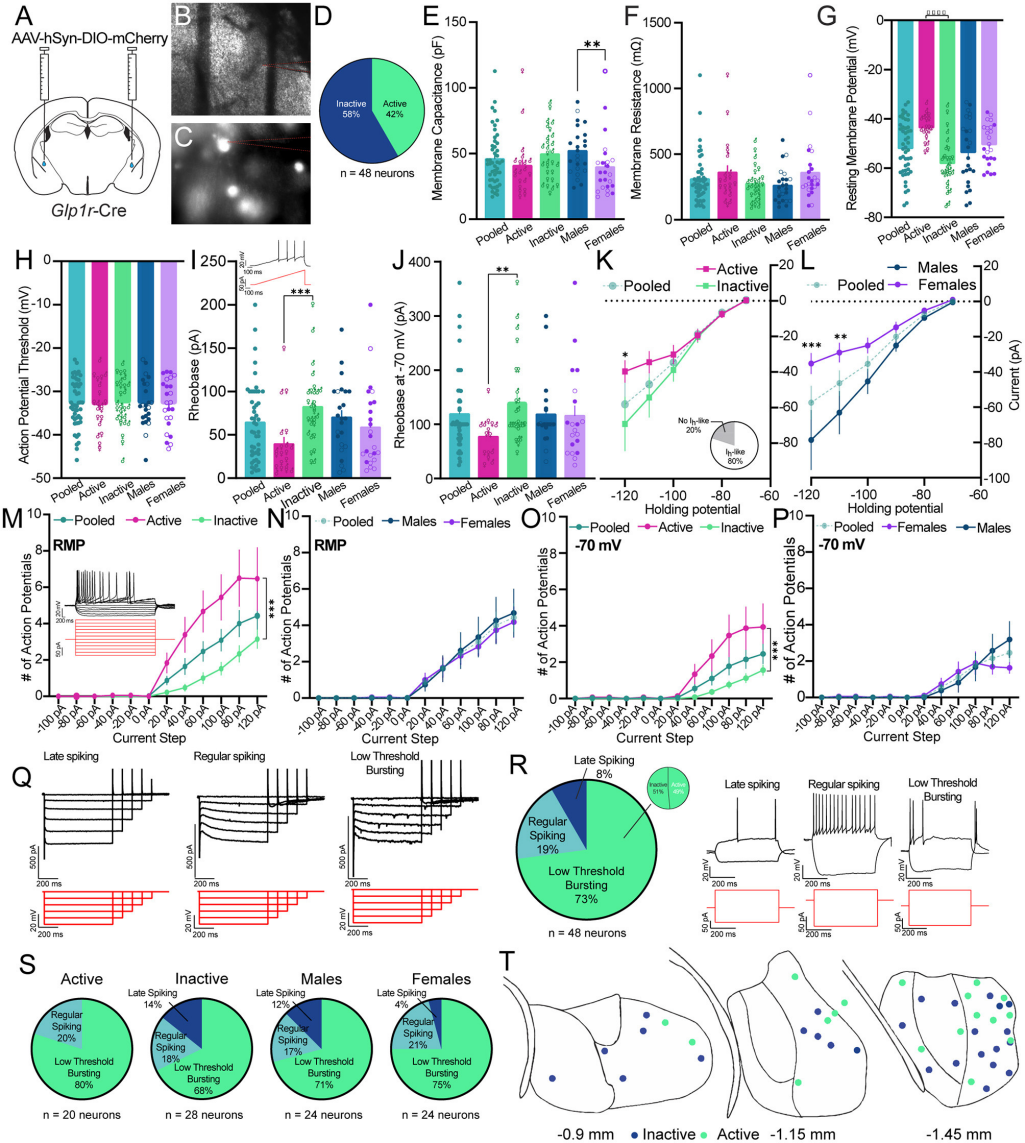
*Ex vivo* patch clamp electrophysiological characterization of *Glp*1*r*^CeA^ neurons. **(A)** Schematic of CeA injection of pAAV-hSyn-DIO-mCherry in *Glp1r*-Cre mice. **(B,C)** Example images of live brain slices and fluorescently labeled cells in the CeA during electrophysiological recordings from *Glp1r*-Cre mice. **(D)** Pie chart illustrating the number of *Glp*1*r*^CeA^ neurons that were active/inactive. **(E)** Average membrane capacitance (Cm) of *Glp*1*r*^CeA^ neurons in all cells (pooled) (teal), in neurons empirically determined to be active (pink) or inactive (green), or in neurons recorded from male (blue) or female (purple) mice. Within the active/inactive data sets the sex of the mouse from which the neuron was recorded is depicted by use of male and female symbols. Within the male/female data sets the activity state of the neuron is indicated by a closed (inactive) or open (active) symbol. Data were analyzed using a Mann-Whitney *U* test between active/inactive (*p* = 0.0736) and male/female (*p* = 0.0063) subgroups. **(F)** Average membrane resistance (Rm) of *Glp*1*r*^CeA^ neurons in all cells (pooled) or active, inactive, male, or female subgroups. The symbol conventions used are identical to those used in **(E)**. Data were analyzed using a Mann-Whitney *U* test between active/inactive (*p* = 0.2296) and male/female (*p* = 0.0609) subgroups. **(G)** Average resting membrane potential (Vrest) of all *Glp*1*r*^CeA^ neurons (pooled), or active, inactive, male, or female subgroups. The symbol conventions used are identical to those used in **(E)**. Data were analyzed using a Mann Whitney *U* test between active/inactive (*p* < 0.0001) and male/female (*p* = 0.4677) subgroups. **(H)** Average action potential threshold of all *Glp*1*r*^CeA^ neurons (pooled), or active, inactive, male, or female subgroups. The symbol conventions used are identical to those used in **(E)**. Data were analyzed using a Mann-Whitney *U* test between active/inactive (*p* = 0.8319) and male/female (*p* = 0.9796) subgroups. **(I)** Average rheobase of all *Glp*1*r*^CeA^ neurons (pooled), or active, inactive, male, or female subgroups at rest. The symbol conventions used are identical to those used in **(E)**. Data were analyzed using a Mann-Whitney *U* test between active/inactive (*p* = 0.0001) and male/female (*p* = 0.2276) subgroups. Inset: representative cell response in current clamp mode to gradually increasing depolarizing current injection (rheobase). Bottom: Stimulation waveform **(J)** Average rheobase current of all *Glp*1*r*^CeA^ neurons (pooled), or active, inactive, male, or female subgroups at −70 mV. The symbol conventions used are identical to those used in **(E)**. Data were analyzed using a Mann-Whitney *U* test between active/inactive (*p* = 0.0058) and male/female (*p* = 0.4877) subgroups. **(K)** Average I_h_-like current at hyperpolarizing membrane potentials in all, active, or inactive *Glp*1*r*^CeA^ neurons. Active and inactive subgroups were compared using 2-way repeated measures ANOVA with Sidak's multiple comparisons test at each holding potential; Holding potential X Activity state: *F*_(5,215)_ = 2.413, *p* = 0.0373; Holding potential: *F*_(5,215)_ = 28.95, *p* < 0.0001; Activity state: *F*_(1,43)_ = 1.467, *p* = 0.2324. The inset pie chart displays the proportion of neurons that demonstrate significant I_h_-like current using a threshold of −20 pA for the −120-mV voltage step. **(L)** Average I_h_-like current at hyperpolarizing membrane potentials in all, male, or female *Glp*1*r*^CeA^ neurons. Male and female subgroups were compared using 2-way repeated measures ANOVA with Sidak's multiple comparisons test at each holding potential; Holding potential X Sex: *F*_(5,215)_ = 4.976, *p* = 0.0002; Holding potential: *F*_(5,215)_ = 34.80, *p* < 0.0001; Sex: *F*_(1,43)_ = 6.394, *p* = 0.0152. **(M)** Action potentials in response to depolarizing current steps at rest in all, active, or inactive *Glp*1*r*^CeA^ neurons. Active and inactive subgroups were compared using 2-way repeated measures ANOVA with Sidak's multiple comparisons test at each current injection; Current Injected X Activity state: *F*_(11,473)_ = 7.881, *p* < 0.0001; Current Injected: *F*_(11,473)_ = 33.93, *p* < 0.0001; Activity state: *F*_(1,43)_ = 11.83, *p* = 0.0013. Top: representative cell response in current clamp mode to increasing current steps. **(N)** Action potentials in response to depolarizing current steps at rest in all, male, or female *Glp*1*r*^CeA^ neurons. Male and female subgroups were compared using 2-way repeated measures ANOVA with Sidak's multiple comparisons test at each current injection; Current Injected X Sex: *F*_(11,473)_ = 0.2256, *p* = 0.9958; Current Injected: *F*_(11,473)_ = 24.96, *p* < 0.0001; Sex: *F*_(1,43)_ = 0.07738, *p* = 0.7822. **(O)** Action potentials in response to depolarizing current steps at −70 mV in all, active, or inactive *Glp*1*r*^CeA^ neurons. Active and inactive subgroups were compared using 2-way repeated measures ANOVA with Sidak's multiple comparisons test at each current injection; Current Injected X Activity state: *F*_(11,418)_ = 5.822, *p* < 0.0001; Current Injected: *F*_(11,473)_ = 19.79, *p* < 0.0001; Activity state: *F*_(1,38)_ = 8.353, *p* = 0.0063. **(P)** Action potentials in response to depolarizing current steps at −70 mV in all, male, or female *Glp*1*r*^CeA^ neurons. Male and female subgroups were compared using 2-way repeated measures ANOVA with Sidak's multiple comparisons test at each current injection; Current Injected X Sex: *F*_(11,418)_ = 1.358, *p* = 0.1903; Current Injected: *F*_(11,418)_ = 14.02, *p* < 0.0001; Sex: *F*_(1,38)_ = 0.05488, *p* = 0.8160. **(Q)** Example I_h_-like currents across three observed spiking subtypes for *Glp*1*r*^CeA^ neurons. The capacitive currents have been cropped for visualization purposes. Voltage step waveforms are shown below each cell. **(R)** Pie chart illustrating the distribution of spiking subtypes (late spiking, regular spiking, or low-threshold bursting) for *Glp*1*r*^CeA^ neurons in response to hyperpolarizing or depolarizing current steps. Example late spiking, regular spiking and low-threshold bursting *Glp*1*r*^CeA^ neurons in response to depolarizing current steps are shown to the right. **(S)** Pie charts illustrating the distribution of spiking subtypes among inactive, active, male, or female subgroups. The distributions of active/inactive and male/female subgroups were compared using a Chi-squared test for trend (*p* = 0.1511 for active/inactive and *p* = 0.4911 for male/female). **(T)** Schematic of distribution of recorded *Glp*1*r*^CeA^ neurons. Blue dots represent inactive and green dots represent active neurons. *N* = 48 neurons from male (*n* = 10) and female (*n* = 7) mice with 24 neurons per sex.

To gain insights into the functional nature of *Glp*1*r*^CeA^ cells, we used *ex vivo* brain slices combined with whole cell patch clamp electrophysiology. We injected male and female *Glp1r*-Cre mice with AAV-DIO-mCherry and waited 3–4 weeks prior to preparing fresh brain slices (Figures 3A,B). *Glp*1*r*^CeA^ neurons were identified using DIC and epifluorescence (Figure 3C). On average, *Glp*1*r*^CeA^ neurons had a membrane capacitance (Cm) of 45.75 ± 3.03 pF and a membrane resistance (Rm) of 309 ± 26.9 mΩ (Figures 3E,F). In gap free current clamp mode, we measured the average resting membrane potential (RMP) and excitability of *Glp*1*r*^CeA^ neurons. Interestingly, we observed that 42% of *Glp*1*r*^CeA^ neurons are spontaneously active at rest (Figure 3D) and had an average RMP of −51.93 ± 1.59 mV (Figure 3G). Spontaneously active *Glp*1*r*^CeA^ neurons displayed an average firing frequency of 0.47 Hz. To understand if the activity state of *Glp*1*r*^CeA^ neurons is associated with neurophysiological characteristics, we analyzed the Cm, Rm, and RMP within both active and inactive *Glp*1*r*^CeA^ neuron subgroups. Consistent with their activity states, active neurons had a higher RMP than inactive neurons (active = −43.48 ± 1.217 mV vs. inactive = −57.97 ± 1.878 mV), but we observed no differences in Cm (active = 40.66 ± 5.039 pF vs. inactive = 49.39 ± 3.681 pF) or Rm (active = 360.5 ± 53.98 mΩ vs. inactive = 272.3 ± 23.99 mΩ) (Figures 3E–G). We also quantified differences in basal neuronal properties in male vs. female *Glp*1*r*^CeA^ neuron subgroups. Surprisingly, we observed a sex difference in the Cm of *Glp*1*r*^CeA^ neurons, where *Glp*1*r*^CeA^ neurons from female mice (39.56 ± 4.584 pF) had a significantly lower Cm than male from male mice (51.95 ± 3.646 pF) (Figure 3E). We observed no statistically significant differences in the Rm (male = 260.1 ± 26.51 mΩ vs. female = 358 ± 45.25 mΩ) or RMP (male = −53.53 ± 2.691 mV vs. female = −50.34 ± 1.679 mV) between male and female mice (Figures 3E,G).

Using whole cell patch clamp electrophysiology, we probed other neurophysiological phenomena that typify CeA neurons. On average, *Glp*1*r*^CeA^ neurons show I_h_-like currents (Figures 3K,L,Q) though we observed that 20% of *Glp*1*r*^CeA^ neurons showed no detectable I_h_-like currents (inset in Figure 3K). We quantified I_h_-like currents in both active and inactive neurons and observed a statistically significant reduction in I_h_-like currents in active neurons that was most evident at a hyperpolarized potential of −120 mV (Figure 3K). Similarly, we observed a significant reduction in I_h_-like current in *Glp*1*r*^CeA^ neurons from female mice relative to male mice that was evident at multiple holding potentials (Figure 3L).

In response to ramping positive current injections in current clamp mode, *Glp*1*r*^CeA^ neurons demonstrated action potentials with an average threshold of −32.8 ± 0.800 mV (Figure 3H). We observed no statistical differences between active (−33.08 ± 1.292 mV) and inactive (−32.64 ± 1.034 mV) or between male (−32.77 ± 1.108 mV) and female (−32.88 ± 1.179 mV) *Glp*1*r*^CeA^ neurons. As nearly half of *Glp*1*r*^CeA^ neurons were spontaneously active, we measured the current needed to produce an action potential (rheobase) at rest and while holding the cell at −70 mV. At rest, the rheobase for *Glp*1*r*^CeA^ neurons was 64.26 ± 6.45 pA whereas at −70 mV the rheobase was 118.8 ± 10.74 pA (Figures 3I,J). At rest, we found that active neurons (39.22 ± 8.305 pA) demonstrate a significantly lower rheobase than inactive neurons (82.15 ± 7.814 pA) (Figure 3I). Interestingly, active neurons (76.99 ± 9.579 pA) displayed a significantly lower rheobase than inactive neurons (140.9 ± 16.24 pA) even when held at −70 mV (Figure 3J). We observed no statistically significant differences in *Glp*1*r*^CeA^ neuron rheobase between male and female mice at rest (males: 69.97 ± 8.487 pA vs. females: 58.56 ± 9.756 pA) (Figure 3I) or at −70 mV (males: 118.3 ± 14.66 pA vs. females: 115.6 ± 18.79 pA) (Figure 3J).

To further characterize *Glp*1*r*^CeA^ neuronal excitability, we measured changes in membrane voltage and action potentials in response to increasingly positive square current steps in *Glp*1*r*^CeA^ neurons held at their resting membrane potential and at −70 mV. In response to current injections, we found that active *Glp*1*r*^CeA^ neurons display significantly more action potentials than inactive *Glp*1*r*^CeA^ neurons at rest (Figure 3M) or at −70 mV (Figure 3O) and the increase in the number of action potentials was most pronounced at the highest positive current injections. We observed no difference in current injection-induced action potential firing between male and female *Glp*1*r*^CeA^ neurons either at rest (Figure 3N) or held at −70 mV (Figure 3P). Forty-one percent of *Glp*1*r*^CeA^ neurons showed spike frequency adaptation at depolarizing current steps (data not shown). Using these data we also classified each neuron according to spike characteristics (Dumont et al., 2002; Chieng et al., 2006; Herman et al., 2013; Li and Sheets, 2018). The majority (73%) of *Glp*1*r*^CeA^ neurons were a low threshold bursting (LTB) subtype with a characteristic rebound action potential that occurs following cessation of a hyperpolarizing current step and low latency action potentials in response to depolarizing current steps (Figure 3R). LTB *Glp*1*r*^CeA^ neurons were equally distributed between active and inactive neurons (inset pie chart in Figure 3R). Moreover, we did not observe a significant difference in the overall distribution of spike types amongst active/inactive or male/female *Glp*1*r*^CeA^ neurons (Figure 3S). The sites of these recordings are presented in Figure 3T.

## Discussion

In this study, we performed the first deep anatomical characterization of *Glp1r* expression and neurophysiology study of *Glp1r*-expressing cells in the CeA of C57BL6/J mice. At an anatomical level, a previous study using a *Glp1r-*Cre mouse strain identified *Glp1r*-expressing cells throughout the brain including the CeA (Cork et al., 2015). Their observations are mirrored by a study from Jensen and colleagues who used a novel GLP-1R antibody and observed GLP-1R protein expression in the CeA (Jensen et al., 2018). In a recent study using a transgenic mouse with an mApple-tagged GLP-1R, the authors rigorously mapped sites of GLP-1R expression throughout the brain and made similar observations, including robust expression in the CeA (Graham et al., 2020). Consistent with their findings using a transgenic mouse, the authors used fluorescence *in situ* hybridization as a convergent method to characterize native *Glp1r* mRNA. They showed that *Glp1r* mRNA is present in the CeA and medial amygdala, and, in the CeA, *Glp1r* is coexpressed with the GABAergic neuronal biosynthetic enzyme *Gad1*. Since *Gad1* is only expressed in neurons, we infer that the *Glp1r* cells we characterized in our study are primarily neuronal.

We observed that *Glp*1*r*^CeA^ neurons are not defined by the sole coexpression with either *Prkcd, Sst*, or *Tac2*. *Prkcd* is a gene that marks a population of lateral and capsular CeA neurons that are activated by aversive unconditioned stimuli, inhibited by aversive conditioned stimuli, and inhibit food intake when activated (Haubensak et al., 2010; Cai et al., 2014; Cui et al., 2017; McCullough et al., 2018a). *Sst* is a neuropeptidergic gene that marks a population of lateral and medial CeA neurons almost completely distinct from *Prkcd* that are activated by aversive conditioned stimuli, modulate passive and active defensive responses, and promote freezing and behavioral cessation when activated using optogenetics (Li et al., 2013; Yu et al., 2016). *Tac2* is a neuropeptidergic gene enriched in medial and some lateral CeA neurons and modulates the expression of conditioned fear (Andero et al., 2014, 2016). A recent study identified that ~50% of *Glp*1*r*^CeA^ neurons coexpress the cytokine interleukin-6 (IL-6) and that direct administration of IL-6 to the CeA could produce a subtle decrease in feeding, but the functional impact of IL-6 in combination with CeA GLP-1Rs is unclear (Anesten et al., 2019). The limited overlap we observed between *Prkcd*/*Sst*/*Tac2* and *Glp1r* in the CeA suggests that the anorexigenic actions of peripherally-applied or intracerebral GLP-1R agonists are likely not due to the direct activation of these neurons, but do not preclude their activation through indirect network effects (Gabery et al., 2020). Further experiments using *in vivo* recordings are necessary to test direct and indirect models of action of GLP-1R agonists on these genetically defined CeA neurons.

Using Cre-dependent viral tracing in combination with *Glp1r*-Cre mice, we performed cell counts of *Glp1r* cells across the rostral-caudal axis and in CeA subnuclei. *Glp*1*r*^CeA^ neurons are located throughout the rostral-caudal axis with a non-significant increase in the middle third of the CeA and are more densely distributed in the CeM. Coincidently, the CeA also receives innervation from Preproglucagon (PPG, the precursor gene of GLP-1)-expressing neurons in the nucleus of the solitary tract and this innervation is most dense in the CeM (Llewellyn-Smith et al., 2011; Williams et al., 2018; Anesten et al., 2019). The functional and behavioral role of PPG inputs to the CeA are unknown.

Using brain slice electrophysiology, we performed a neurophysiological characterization of *Glp*1*r*^CeA^ neurons in male and female mice. Surprisingly, we found that nearly half of these neurons are active at rest. We analyzed neurophysiological characteristics associated with inherent neuronal activity states. Not surprisingly, we found that active *Glp*1*r*^CeA^ neurons have a higher resting membrane potential than inactive neurons and are more sensitive to current injections. Interestingly, the increased sensitivity of active *Glp*1*r*^CeA^ neurons to current injection-induced action potentials persists even when held at an identical polarized membrane potential of −70 mV. As seen in other CeA neuronal physiology studies, *Glp*1*r*^CeA^ neurons on average show hyperpolarization-activated (I_h_) currents; however, we did observe some cells for which no substantial I_h_-like current was detected (20%). Interestingly, we found reduced I_h_-like currents in *Glp*1*r*^CeA^ neurons recorded from female mice and that, on average, these neurons have a lower membrane capacitance. The functional implications of these sex differences is unclear although they may be related to overall morphometric size differences that have been reported in the amygdala (Hines et al., 1992; Qiu et al., 2018). For *Glp*1*r*^CeA^ neurons, we characterized their excitability using current injections in current clamp mode and determined their spiking subtype. Overall, we found that *Glp*1*r*^CeA^ neurons are predominantly a low threshold bursting subtype characterized by rebound spikes from hyperpolarizing current injections. We speculate that cells of the low threshold burst subtype may be particularly sensitive to excitatory input or activation of intracellular signaling pathways that enhance excitability like GLP-1Rs. Several heroic neurophysiological studies of CeA neurons in general or CeL neurons demonstrate a diversity of spiking types (Dumont et al., 2002; Chieng et al., 2006; Hunt et al., 2017; Adke et al., 2021). Only a couple of studies have focused neurophysiological recordings on the CeM, which is more diverse with respect to gene expression and axonal projections (Herman et al., 2013; Li and Sheets, 2018; McCullough et al., 2018b). Interestingly Li and colleagues showed that periaqueductal gray (PAG)-projecting CeA neurons are distributed in the CeL and CeM and did an extensive study of their firing properties and excitability broken down by firing subtype. PAG-projecting CeM neurons are heterogenous and comprised of equal proportions of regular spiking, fast-spiking, and bursting subtype and demonstrate voltage sag in response to hyperpolarizing current injections indicative of I_h_-like currents (Li and Sheets, 2018). However, the authors did not observe sex differences in the voltage sag of PAG-projecting CeM neurons. These data suggest that *Glp*1*r*^CeA^ neurons are likely distinct from CeM-projecting neurons, however further studies using anterograde and tracers are needed to rule out this possibility. CeA neurons that express corticotropin releasing factor receptor 1 (CRFR1 or *Crhr1*) are distributed in the medial and lateral subdivisions of the CeA, and the reported membrane properties, resting membrane potential, and spiking types most closely match those in this study (Herman et al., 2013). CRFR1, like GLP-1R, is a G_αs_-coupled receptor so it will be interesting to know in the future if *Glp*1*r*^CeA^ neurons coexpress *Crhr1*.

GLP-1R agonists are widely prescribed for the treatment of type II diabetes and they also have the added benefit of suppressing appetite and producing weight loss (Drucker, 2018). Recently the FDA approved the use of semaglutide (a new generation GLP-1R agonist) for the treatment of obesity. Unfortunately, the mechanisms by which these drugs exert their anorexigenic effects in the brain is still unclear. We hypothesize that the CeA, in addition to the hypothalamus and hindbrain, is an important limbic nucleus that mediates some of the anorexigenic effects of these drugs. Because the CeA is a nucleus that receives endogenous GLP-1 through preproglucagon inputs from the NTS, we also speculate that that CeA GLP-1Rs may play a role in feeding, emotional, and motivational processing. One study using non-human primates demonstrated that GLP-1Rs are present in the amygdala (Heppner et al., 2015), but further research using post-mortem tissue from humans is needed to demonstrate that GLP-1Rs are expressed in the human amygdala. In addition, basic research using genetic and neural circuit approaches are needed to characterize a circuit logic for the mode of action of GLP-1R agonists.

## Data Availability Statement

The raw data supporting the conclusions of this article will be made available by the authors without undue reservation.

## Ethics Statement

The animal study was reviewed and approved by UAB IACUC.

## Author Contributions

NZ, EC, CL, SK, and MD collected data for the manuscript. NZ and JH analyzed the data. NZ, SV, and JH wrote the manuscript and assembled the figures. JH designed the study. All authors contributed to the article and approved the submitted version.

## Funding

This project was supported by K01DK115902 and R03DK129561 to JH and the UAB Nutrition Obesity Research Center Pilot and Feasibility Program P30DK056336.

## Conflict of Interest

The authors declare that the research was conducted in the absence of any commercial or financial relationships that could be construed as a potential conflict of interest.

## Publisher's Note

All claims expressed in this article are solely those of the authors and do not necessarily represent those of their affiliated organizations, or those of the publisher, the editors and the reviewers. Any product that may be evaluated in this article, or claim that may be made by its manufacturer, is not guaranteed or endorsed by the publisher.
